# Simultaneous detection of the shuttling motion of liquid metal droplets in channels under alternating pressure and capacitive sensor signals

**DOI:** 10.1038/s41378-024-00652-1

**Published:** 2024-03-29

**Authors:** Shinji Bono, Ryotaro Nakai, Satoshi Konishi

**Affiliations:** 1 Research Organization of Science and Technology, Ritsumeikan University, Shiga, Japan; 2 Ritsumeikan Advanced Research Academy, Kyoto, Japan; 3 Ritsumeikan Global Innovation Research Organization, Ritsumeikan University, Shiga, Japan; 4 Graduate School of Science and Engineering, Ritsumeikan University, Shiga, Japan

**Keywords:** Electrical and electronic engineering, Materials science

## Abstract

Implementing a signal-switching mechanism for the selective use of integrated sensors and actuators plays a crucial role in streamlining the functionality of miniaturized devices. Here, a liquid metal droplet (LMD)-based signal-switching mechanism is introduced to achieve such functionality. Pressure modulation with a 100-μm spatial resolution enabled precise control of the position of the LMDs within a channel. After integrating the channel with asymmetrically structured electrodes, the effect of the shuttle-like movement of LMD on the temporal changes in the overall capacitance was investigated. Consequently, analysis of the capacitive peaks revealed the directional movement of the LMDs, enabling estimation of the position of the LMDs without direct observation. In addition, we achieved successful signal extraction from the capacitive sensor that was linked to the activated electrodes, thereby enabling selective data retrieval. The proposed signal-switching mechanism method achieved a detection accuracy of ~0.1 pF. The sensor’s ability to simultaneously detect the LMD position and generate a signal underscores its significant potential for multiplexing in multisensing systems, particularly in concealed environments, such as in vivo settings.

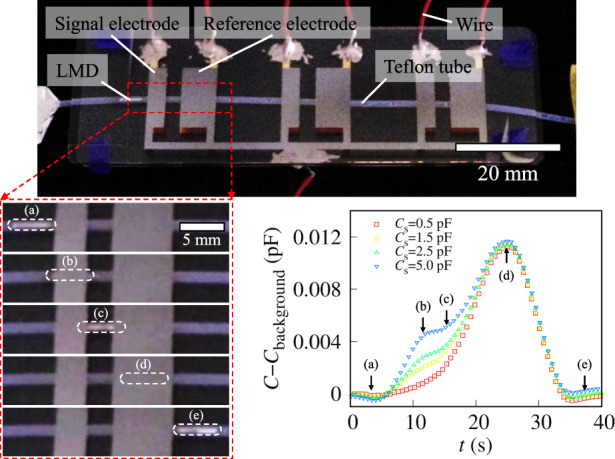

## Introduction

Advances in microelectromechanical system techniques have enabled the miniaturization of sensors and actuators, such that many sensors and actuators can now be integrated into a chip, thereby enabling multiple functionalities^1,2^. An illustrative example involves the incorporation of sensors and actuators into elongated medical instruments and robots. The integration of pneumatic actuators into an endoscope enables a clear endoscopic view due to the exclusion of biological tissue^3,4^. Incorporating pressure sensors into a stent or catheter enables effective and regular monitoring of the health of affected parts while holding a lumen^5,6^. Therefore, the multifunctionalization of medical instruments based on sensor–actuator integration is essential for achieving minimally invasive surgery and regular health monitoring.

As the number of sensors and actuators on a chip increases, complex wiring and structures become increasingly challenging. To simplify these wirings and structures, the use of multiplexers with a signal-switching mechanism (SSM) is advantageous. Therefore, SSM-incorporated liquid metal droplets (LMDs) have been developed^7^. Conventional LMDs include mercury^8,9^ because its fluidity and electroconductivity are high, which is appropriate for SSM. However, mercury is highly toxic and has a heavy environmental load, necessitating its restriction of use. In contrast, gallium alloy (Galinstan) is a promising alternative material to mercury^10–12^ because it offers comparable fluidity and electroconductivity along with low toxicity.

A Galinstan oxide layer formed spontaneously on the LMD surface under atmospheric exposure^13^. The tendency of the oxide layer to adhere to the channel wall decreased the LMD fluidity. We previously suppressed this adhesion by introducing continuous fluids alongside LMDs, which resulted in the successful and smooth transport of LMDs within a single channel^14^.

As the oxide layer exhibits significantly lower electroconductivity than bulk Galinstan, alternating current (AC), but not direct current signals, is commonly employed for Galinstan-based LMD-incorporated SSMs without special treatment to remove the oxide layer^7,15^. AC signals can pass through the oxide layer with finite capacitance. Previously, we introduced an LMD surrounded by a continuous fluid into a flexible channel sandwiched by a pair of serially arranged electrodes^7^. Insertion of an LMD between electrodes decreases the impedance of the electrode pair. Therefore, it is feasible to selectively acquire electrical signals from sensors linked to activated electrodes through impedance variation.

To obtain signals from the integrated sensors, we unidirectionally transported the LMDs unidirectionally. Thus, the LMD method scans serially arranged electrode pairs continuously and unidirectionally. Therefore, selective access of LMDs to the target sensor was unavailable; that is, continuous scanning using LMDs has no spatial resolution. To improve both the spatial resolution and accessibility of target sensors, intensive variables such as pressure are required to regulate the LMD position in a channel. If this variable serves as the control parameter for the LMD position, activated sensors can be selected based on this control parameter.

Identifying both the control parameters and LMD position is crucial. Although direct observation of LMDs can enable the determination of activated sensors, the use of activated sensors for in vivo medical applications, such as on stents, remains difficult^16^ because such activated sensors cannot be identified even though signals may be available. In applications, in vivo multisensing systems require the capability to identify which sensors output the signals without direct observation.

In this study, we use pressure to drive the shuttling motion of an LMD in a channel and selectively acquire capacitive sensor signals. Because pressure sensors enable regular monitoring of affected parts, integrating capacitive sensors into SSMs is crucial. However, distinguishing capacitive sensor signals from the capacitances of electrode pairs is challenging. To overcome this problem, we designed reference and signal electrodes with asymmetric geometry and applied pressure to LMD in a flexible tube integrated with the electrodes. First, we quantitatively investigated the pressure–LMD position relationship, followed by spatial resolution estimation. The total capacitance of the LMD device during shuttle-like movement was subsequently measured. The proposed mechanism enables concurrent detection of the LMD position and capacitive signals from the sensors.

## Results

### Concept of our sensor-switching mechanism

**Fig. 1:**
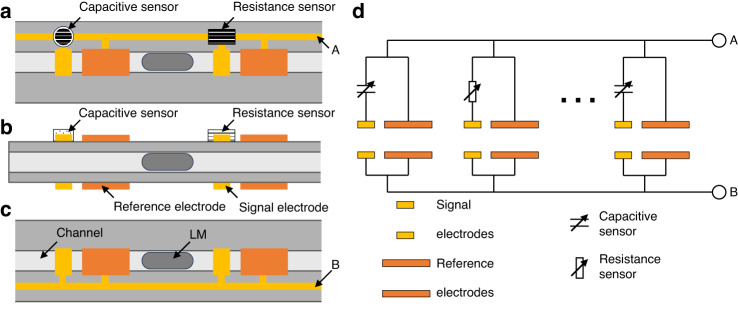
Conceptual image of the sensor-switching mechanism (SSM) using the shuttling motion of liquid metal droplets (LMDs). **a** Schematic top view, **b** side view, and **c** bottom view of the switching mechanism. **d** Equivalent circuit of the SSM. We connected the sensors and signal electrodes to serially arranged electrodes

Figure 1 shows the concept of our SSM using LMD. A channel was sandwiched between a sequence of paired electrodes, and multiple sensors were connected to these electrodes. The system selectively acquires sensor signals based on the pressure-regulated LMD position. The electrodes were patterned as shown in Fig. 1a–c. Figure 1d shows the equivalent circuit of the switching device. Prior studies have shown that LMD insertion between electrodes decreases paired electrode impedance^7,14^. Therefore, we selectively access the sensor linked to the activated electrode. Changes in electrical properties are commonly used as sensor signals. We focused on capacitive and resistance sensors, which have been previously reported^7^.

For the simultaneous detection of LMD positions within a channel and sensor signals, pairs of reference and sensor electrodes were incorporated. Notably, the electrode design features asymmetry, which results in the initial capacitance of the signal electrodes being lower than that of the reference electrodes. Moving to the right (or left), the LMD electrode passes the reference (signal) electrode after the signal (reference) electrode. The capacitance of the activated electrodes increased with increasing amplification factor^14^. Therefore, when the LMD moves to the right (left), small (large) signals follow large (small) signals. When the impact of capacitive sensors is less significant than the offset, distinguishing the direction of the LMD shuttle motion becomes feasible through temporal changes in the capacitive signals. A concurrent detection mechanism for capacitive signals and activated sensors was established.

### Time evolution of capacitance without sensor signals during LMD transport

**Fig. 2:**
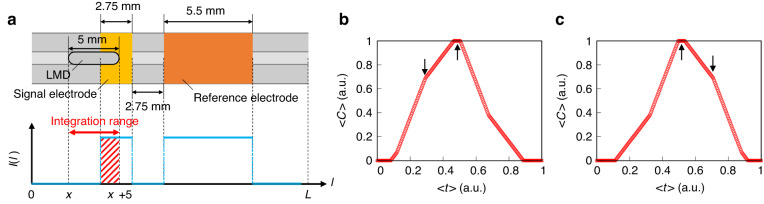
Numerical calculation for estimating the switching device capacitance during LMD shuttling motion. **a** Schematic illustration of the calculation geometry and indicator function. The red arrow and blue line show the integration area (*x* < *l* < *x* + 5) and the indicator function, respectively. The area filled by the red lines represents the increase in the total capacitance Δ*C*(*x*). The time evolution of the normalized capacitance $\langle C\rangle$ under **b** compression and **c** decompression. The black arrows indicate of the $\langle C\rangle$ *peaks*

To estimate the temporal evolution of the capacitance of the switching device, we performed a numerical analysis. Figure 2a shows the conditions of the numerical calculations. We define the axis along the channel as the *l*-axis. The widths of the LMD and electrodes corresponded to the experimental values. The LMD moves within 0 ≤ *x* ≤ *l*_max_, where *l*_max_ is the interval between the signal electrodes. LMD shuttles with a constant velocity *v*.

When LMD enters between a pair of electrodes, the capacitance of the electrode pair increases. We assumed that the increase in the capacitance was proportional to the overlap length^14^. We define the amplification factor of the capacitance per unit length as δ*c*. The increase in the total capacitance Δ*C*(*x*) is given as follows:

1a $$\varDelta C(x)=\delta c{\int }_{x}^{x\,+\,5}I(l\,){\rm{d}}l$$

1b $$I(l)=\left\{\begin{array}{cc}1 & {\rm{LMD}}\,\in \,{\rm{electrodes}}\\ 0 & {\rm{LMD}}\,\notin \,{\rm{electrodes}}\end{array}\right.$$

where *I*(*l*) is the indicator function. The integration range of Eq. (1a) is the area occupied by LMD. Δ*C*(*x*) is the area indicated by the red lines in Fig. 2a. We calculated Δ*C*(*x*) numerically.

Figure 2b shows the time evolution of the capacitance for *v* > 0. We normalize Δ*C*(*x*) and *t* as follows:

2a $$\langle C\rangle =\frac{\varDelta C}{5\delta c}$$

2b $$\langle t\rangle =\frac{|v|t}{L}$$

respectively. We observed two $\left\langle C\right\rangle$ peaks in Fig. 2b, where the first peak was smaller than the second peak.

We show the numerical result of $\left\langle C\right\rangle$ with *v* < 0 in Fig. 2c. The inversion of *v* reverses the relationship between the peaks. Thus, the direction of movement can be identified based on the asymmetric temporal profiles of the evolving capacitance.

### Spatial control of LMD by pressure

**Fig. 3:**
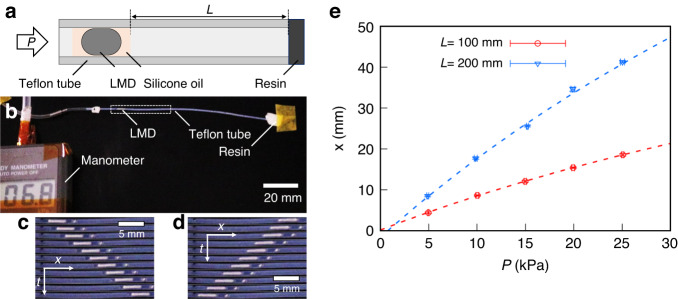
Spatial control of LMD in a channel by pressure. **a** Schematic and **b** photograph of the experimental system. We set the initial length of the volume control space *L*. Spatiotemporal diagrams showing **c** compression [Supplementary Video 1 (SI)] and **d** decompression [Supplementary Video 2 (SI)]. We constructed diagrams from snapshots taken every 1 s. **e** Pressure *P* dependence of the LMD distance, *x*. The dashed lines are the best fits obtained using Eq. (3). The obtained fitting parameters (*A*, *B*) are 9.3 ×10 mm, 9.4 ×10^3^ m Pa and 2.1 ×10^2^ mm, 2.2 ×10^4^ m Pa for *L* = 100 mm and 200 mm, respectively

Figure 3a shows a schematic of the experimental setup for the spatial control of LMD in a channel. We introduced silicone oil with LMD to prevent adhesion of the Galinstan oxide layer. A cylindrical flexible tube is used as the channel.

We sealed one side of the tube with resin and set a volume control space comprising air between the silicone oil and the sealed side. We define the length of the volume control space as *L*. Applying pressure from the opposite side of the sealed side (i.e., the open side) compresses the air in the volume control space. The LMD position was regulated through compression and decompression.

Figure 3b shows a photograph of the experimental system. Pressure *P* (=0 ~ 25 kPa) was applied to the tube using a syringe pump from the left, and the pressure in the tube was monitored using a manometer. The LMD position was quantitatively measured as a function of *P* under the initial conditions of *L* = 100 mm and 200 mm.

During compression, the LMD signal enclosed by the dashed line in Fig. 3b was observed. Figure 3c shows a spatiotemporal diagram constructed by 1-s interval snapshots of LMD under the initial condition of *L* = 200 mm. This compression resulted in LMD transport from the open side to the sealed side of the tube. However, upon decompressing the volume control space, the LMD directional movement reverses (Fig. 3d): the LMD moves from the sealed side to the open side. Because we changed the pressure at a constant rate, LMDs were transported at a constant velocity (~1.5 mm s^−1^) under compression and decompression, as shown in Fig. 3c, d. Overall, these findings indicate that the directional movement of LMD can be controlled by pressure.

Next, we show the *P* dependence of the distance from the initial position *x* in Fig. 3e. In this case, *x* monotonously increased with increasing *P*. The slope $(=\frac{{\rm{d}}x}{{\rm{d}}{P}})$ increased with increasing *L*. The experimental results were fitted using the following equation:

3 $$x=A+\frac{B}{{P}_{0}\,+\,P}$$

where *P*_0_ = 101.3 kPa is the atmospheric pressure. The fitting parameters (*A*, *B*) are 9.3 × 10 mm, 9.4 × 10^3^ m Pa and 2.1 × 10^2^ mm, 2.2 × 10^4^ m Pa for *L* = 100 mm and 200 mm, respectively. These results suggest that the LMD position within a channel can be controlled by pressure through compression and decompression of the volume control space.

### Detection of capacitive sensor signals

**Fig. 4:**
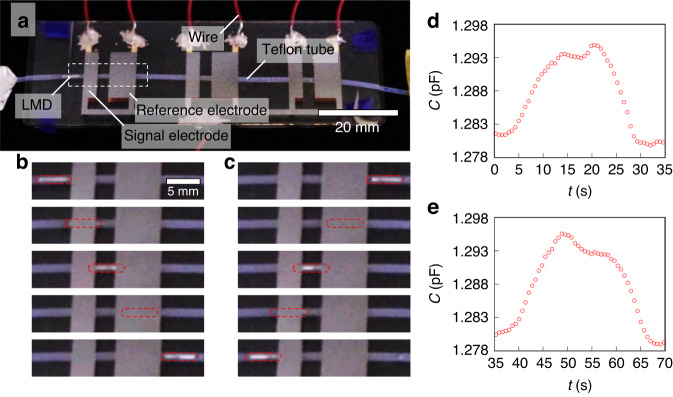
Detection of the time evolution of capacitance with respect to the shuttling motion of LMD. **a** Perspective photo of the switching device comprising a channel and electrodes. Snapshots of LMD under **b** compression [Video 3 (SI)] and **c** decompression [Video 4 (SI)]. We have highlighted the LMDs with red dashed squares. Time evolution of the capacitance under **d** compression and **e** decompression

We integrated the channel and three pairs of reference and signal electrodes and subsequently estimated the electrical properties of the switching device. Figure 4a shows the LMD in the switching device, where the widths of the reference and signal electrodes are 5.50 and 2.75 mm, respectively. To estimate the electrical properties of the device, the total capacitance was investigated by disregarding the sensors.

While the LMDs were driven in a controlled motion with a consistent velocity, *v*, we monitored the change in capacitance of the switching device over time. Figure 4b shows time-dependent snapshots of the LMD in the device during compression. The LMD was initially located to the left of the signal electrode, later overlapped with the signal electrode, and finally was found between the signal and reference electrodes. After passing through the reference electrode, the LMD was transported to the right of the reference electrode. Decompression of the system reversed this motion (Fig. 4c).

Figure 4d, e shows the temporal changes in capacitance during compression and decompression, respectively. Two distinct capacitance peaks were observed: smaller and larger peaks corresponded to situations in which the LMD coincided with the signal and reference electrodes, respectively. These dynamics occur because the reference electrode is wider than the signal electrode. The first peak was smaller (larger) than the second peak under compression (decompression). These results agree with the numerical calculations shown in Fig. 2b, c. Thus, the time evolution profile of the capacitance corresponds to the directional movement of the LMD; that is, we can estimate the direction of movement. For example, if the LMD moves right twice, then it must reside between the second reference electrode and the third signal electrode. Therefore, we can identify which signal electrode LMD is accessing without direct observation.

Previously, LMDs were transported unidirectionally at a constant velocity (~0.4 mm s^−1^), and the length scale of serially arranged electrode pairs was similar to that used in this study^7^. Thus, the switching time scale in the previous work was 50 s. Additionally, we transported LMDs by using pressure in this study, and as a result, we obtained a fast transport velocity (1–2 mm s^−1^). Thus, our SSM achieves high-speed switching (~10 s), which is five times faster than that in previous work. To improve the switching time scale, both a fast transport velocity and miniaturization of electrodes are important.

**Fig. 5:**
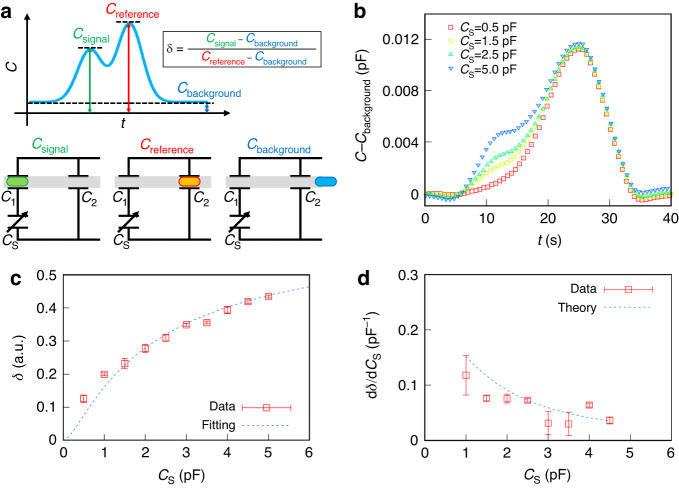
Detection of capacitive sensor signals *C*_S_ based on SSM using LMD. **a** Schematic time evolution of the capacitance and equivalent circuit of the switching device. **b** Time evolution of the normalized capacitance of the SSM with respect to the capacitive sensor. The LMD under compression reaches the signal and reference electrodes at 10 s and 25 s, respectively. We modulated the *C*_S_ to 0.5 pF < *C*_S_ < 5.0 pF. **c** Normalized ratio of the signal capacitance to the reference capacitance δ as a function of the *C*_S_. The dashed line represents the best-fit curve obtained using Eq. (9). The fitting parameter α is 4.3. **d** *C*_S_ dependence of the detection accuracy $\frac{{\rm{d}}\delta }{{\rm{d}}{C}_{{\rm{S}}}}$. The dashed line represents the theoretical prediction from Eq. (10)

Furthermore, we connected the switching device to a capacitive sensor to detect the signals from the capacitive sensor using the LMD shuttling motion. Capacitors with capacitance *C*_S_ were used as capacitive sensors. We then investigated the total capacitance of the switching device as a function of the *C*_S_. When LMD enters between a pair of electrodes, the capacitance increases. Figure 5a shows a schematic of the time evolution of the capacitance. We defined the capacitances with the LMD outside the electrodes, between the signal electrodes, and between the reference electrodes as *C*_background_, *C*_signal_, and *C*_reference_, respectively. Here, *C*_background_ « *C*_signal_ < *C*_reference_.

We connected capacitive sensors to the SSM and subsequently measured the capacitive signals while applying pressure to LMD; that is, LMD passes through reference electrodes after signal electrodes. Here, to remove the effect on the background, we normalize the capacitance to the *C*−*C*_background,_ as shown in Fig. 5b. We observed two capacitance peaks at 10 s and 25 s; the first and second peaks correspond to signals from the signal electrodes and reference electrodes, respectively. In the first scan with *C*_S_ = 0.5 pF, the first peak disappears. Then, we increased the *C*_S_ to 1.5 pF for the second scan. As a result, the signal electrode’s peak increases with the intact reference electrode’s peak. We found that the signal electrode’s peak monotonically increases with increasing *C*_S_, which shows that the SSM enables us to scan capacitive sensors.

We further calculated the normalized ratio of the signal capacitance to the reference capacitance:

4 $$\delta =\frac{{C}_{{\rm{signal}}}-\,{C}_{{\rm{background}}}}{{C}_{{\rm{reference}}}\,-{{\rm{C}}}_{{\rm{background}}}}$$

Figure 5c shows the dependence of the *C*_S_ on *δ*. We found that δ monotonically increases in terms of the *C*_S_. Thus, we can estimate the signal output from using capacitive sensors from δ. To estimate the detection resolution $\left\langle {C}_{{\rm{S}}}\right\rangle$, we calculated the differential $(=\frac{{\rm{d}}\delta }{{\rm{d}}{C}_{{\rm{S}}}})$ using the experimental results in Fig. 5c. Figure 5d shows $\frac{{\rm{d}}\delta }{{\rm{d}}{C}_{{\rm{S}}}}$ as a function of the *C*_S_.

## Discussion

### Spatial resolution of LMD under pressure

We controlled the LMD position by compressing the volume control space under pressure application. The equation of state of an ideal gas in the volume control space is given as follows:

5a $$({P}_{0}+P)V=N\,{k}_{{\rm{B}}}T$$

5b $$V=S(L-x)$$

where *N*, *k*_B_, *T*, *V*, and *S* are the number of molecules in the volume control space, Boltzmann constant, temperature, volume, and cross-sectional area of the channel, respectively. Substituting Eq. (5b) into Eq. (5a), the *P* dependence of *x* is expressed as follows:

6 $$x=L-\frac{N{k}_{{\rm{B}}}T}{S}\frac{1}{{P}_{0}+P}$$

Substituting *k*_B_ = 1.38 × 10^−23^, *T* = 300 K, and *S* ~ 20 mm^2^ into Eq. (6), we obtain $\frac{N{k}_{{\rm{B}}}T}{S}$ ~ 1.1 × 10^4^ m Pa (*L* = 100 mm) and ~2.2 × 10^4^ m Pa (*L* = 200 mm). The fitting parameters *Α* and *Β* in Eq. (3) correspond to *L* and $\frac{N{k}_{{\rm{B}}}T}{S}$, respectively. In our experiment, we obtained *B* = $\frac{N{k}_{{\rm{B}}}T}{S}$ = 9.4 × 10^3^ m Pa for *L* = 100 mm and 2.2 × 10^4^ m Pa for *L* = 200 mm. Our theoretical prediction is consistent with the experimental results, which indicate that the LMD position can be controlled using Eq. (6).

We then defined the spatial resolution of the position control as follows:

7 $$\langle x\rangle ={\frac{{\rm{d}}x}{{\rm{d}}P}\big|}_{P=0}\langle P\rangle =\frac{N{k}_{{\rm{B}}}T}{S{P}_{0}^{2}}\langle P\rangle$$

where $\left\langle P\right\rangle$ is the resolution of the pressure control. Substituting $\left\langle P\right\rangle$ ~ 0.1 kPa and *L* = 100 mm into Eq. (7), we found that our position control of LMD possesses a high spatial resolution $\left\langle x\right\rangle$ ~ 1 × 10^2^ μm.

Previously, the position of LMD could not be identified without direct observation because LMD was continuously transported. However, we succeeded in controlling the position of LMD with high $\left\langle x\right\rangle$ by using pressure. In addition, the interval between the serially arranged electrodes must be larger than $\left\langle x\right\rangle$ to access the target electrode selectively. Thus, $\left\langle x\right\rangle$ corresponds to the limitation of electrode integration. In principle, $1/\left\langle x\right\rangle$ ~ 10 electrodes/mm can be integrated.

### Detection of signals from capacitive sensors using LMD’s shuttling motion

Here, we discuss δ, which represents the theoretical basis of the equivalent circuit of the SSM. The capacitances of the signal and reference electrodes without LMD are denoted as *C*_1_ and *C*_2_, respectively. Their values in the experiment were found to be 0.43 and 0.66 pF, respectively. A previous study reported that LMD insertion increases the electrode capacitance by *α* ( = 1–6) times^14^.

We consider the combined capacitances of the equivalent circuits in Fig. 5a. *C*_background_, *C*_signal_, and *C*_reference_ are expressed as follows:

8a $$\,{C}_{{\rm{background}}}=\frac{{C}_{1}{C}_{{\rm{S}}}+{C}_{{\rm{S}}}{C}_{2}+{C}_{2}{C}_{1}}{{C}_{1}+{C}_{{\rm{S}}}}$$

8b $${C}_{{\rm{signal}}}=\frac{\alpha {C}_{1}{C}_{{\rm{S}}}+{C}_{{\rm{S}}}{C}_{2}+\alpha {C}_{2}{C}_{1}}{\alpha {C}_{1}+{C}_{{\rm{S}}}}$$

8c $${C}_{{\rm{reference}}}=\frac{{C}_{1}{C}_{{\rm{S}}}+\alpha {C}_{{\rm{S}}}{C}_{2}+{C}_{2}{C}_{1}}{{C}_{1}+{C}_{{\rm{S}}}}$$

respectively. Substituting Eqs. (8a), (8b), and (8c) into Eq. (4), δ is given as follows:

9 $$\delta =\frac{{C}_{1}{C}_{{\rm{S}}}^{2}}{{C}_{2}}\frac{1}{({C}_{1}+{C}_{{\rm{S}}})(\alpha {C}_{1}+{C}_{{\rm{S}}})}$$

We fitted the experimental results using Eq. (9) and present the best-fit curve given by the dashed line in Fig. 5c. The obtained fitting parameters (*α* ~ 4.3) agree with previous values^14^. The agreement between our theoretical prediction and experimental results indicates that the capacitive sensor signal can be estimated from *δ*.

Next, we discuss the detection resolution. *δ* is the monotonic function of the *C*_S_, as shown in Eq. (9). Differentiating Eq. (9) with respect to *C*_S_, $\frac{{\rm{d}}\delta }{{\rm{d}}{C}_{{\rm{S}}}}$ is theoretically expressed as follows:

10 $$\frac{{\rm{d}}\delta }{{\rm{d}}{C}_{{\rm{S}}}}=\frac{{C}_{1}^{2}}{{C}_{2}}\frac{(1+\alpha ){C}_{{\rm{S}}}^{2}+2\alpha {C}_{1}{C}_{{\rm{S}}}}{{({C}_{{\rm{S}}}+{C}_{1})}^{2}{({C}_{{\rm{S}}}+\alpha {C}_{1})}^{2}},$$

which corresponds to the detection resolution.$\frac{{\rm{d}}\delta }{{\rm{d}}{C}_{{\rm{S}}}}$ is maximized at *C*_S_ ~ *C*_1_. Thus, to achieve high detection resolution, signal electrodes should be designed such that *C*_1_ equals the typical sensor capacitance.

Note that Eq. (10) is indicated by the dashed line in Fig. 5d. The theoretical predictions agreed with the experimental results.$\frac{{\rm{d}}\delta }{{\rm{d}}{C}_{{\rm{S}}}}$ reaches the maximum value at a *C*_S_ of ~ 0.5 pF. Assuming that the average capacitance of common pressure sensors is ~1 pF, we find that $\left\langle {C}_{{\rm{S}}}\right\rangle$ ~ 0.1 pF, where $\frac{{\rm{d}}\delta }{{\rm{d}}{C}_{{\rm{S}}}}$ ~ 0.15 pF^−1^ and $\langle \delta \rangle$~0.02. Therefore, our proposed SSM using LMD shuttling motion has high detection accuracy.

## Conclusion

Here, we have succeeded in simultaneously detecting the pressure-driven shuttling motion of LMDs and corresponding capacitive sensor signals. The LMD shuttling motion can be generated by compressing the volume control space through pressure modulation in a channel. The application of pressure to the volume control space enables us to serially scan the arranged electrode pairs continuously, repeatedly and selectively. Our pressure-based positional control mechanism exhibits a high spatial resolution (~1 × 10^2^ μm). After the signal and reference electrodes were integrated into a channel, the total capacitance of the device with respect to LMD transport behavior was measured. Consequently, our findings reveal that both the LMD position and movement direction can be deduced from asymmetric temporal changes in capacitance, even without direct observation, thereby underscoring our findings’ significance in the in vivo applications of SSMs.

We obtained signals from a capacitive sensor linked to a signal electrode while driving the LMD shuttling motion. The total capacitance of the device was found to depend on the LMD position, and the *C*_S_ of the capacitive sensor could be estimated with a high detection accuracy (~0.1 pF) based on δ. Our theoretical model, which invokes an equivalent circuit representation, quantitatively agrees with the experimental results. The proposed method could simultaneously detect the LMD position and capacitive signals.

While this study employed capacitive sensors, other sensor types (such as resistance and electromotive sensors) could be connected to the signal electrodes. For example, previous research connected serially arranged electrodes with variable resistances to strain sensors, successfully enabling signal switching from resistance sensors in the order of the LMD passage^7^. Thus, our proposed SSM has potential for direct application in multisensing systems. Operating as a multiplexer, our LMD-based SSM has valuable applicability in unobservable environments, including in vivo scenarios.

## Materials and methods

### Fabrication process of the switching device

**Fig. 6:**
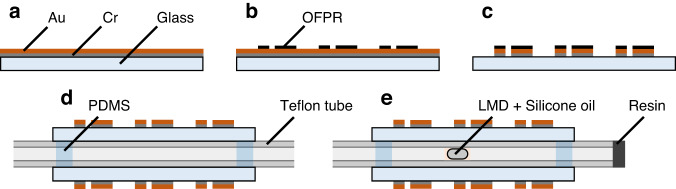
Fabrication process of the switching device. **a** Cr/Au layer formed by thermal evaporation. **b** Photoresist patterning using photolithography. **c** Wet etching of the Cr/Au layer. **d** Integration of a tube and electrode-patterned substrates. **e** Introduction of LMD and silicone oil, with one side of the tube resin sealed

To fabricate the reference and signal electrodes, a 25-nm thick Cr layer and 50-nm thick Au layer were deposited on glass substrates by thermal evaporation (Fig. 6a). We patterned a photoresist (OFPR-800LB, Tokyo Ohka Kogyo Co.) layer on the Au layer using a photolithography technique (Fig. 6b). After partial removal of Au and Cr by wet etching (Fig. 6c), the photoresist was washed out with an organic solvent and then sandwiched in a Teflon tube (TUF-100, Chukoh Chemical Industries, Ltd.) with electrode-patterned substrates (Fig. 6d). The outer and inner diameters of the tube were 1.0 mm and 0.5 mm, respectively. The tube and substrates were then fixed with polydimethylsiloxane (PDMS; SILPOT^TM^ 184; DuPont Toray Specialty Materials K.K.). After introducing Galinstan (ZZGS22011, Zairyo-ya.com) as an LMD and silicone oil (KF-96-50CS, Shin-Etsu Chemical Co., Ltd.) into the tube, one side of the tube was sealed with resin (Super X No. 8008, Cemedine Co., Ltd.) (Fig. 6e).

To improve pressure controllability, we used a Teflon tube that has a low degree of air leakage. A channel composed of common materials such as PDMS can be used to construct an SSM. However, porous materials such as PDMS have high air leakage, which decreases pressure controllability. Thus, an additional mechanism to suppress air leakage is urgently needed in future work.

### Measurement methods

To exert pressure on the channel, we established a connection between the open end of the tube and a syringe pump (YSP-201, YMC.CO., Ltd.), and controlled the pressure in the channel by applying air from the syringe pump to the tube at a constant rate (20–30 mL/min). The inner pressure was monitored using a manometer (PG-100, NIDEC COMPONENTS CORPORATION). We also confirmed that the compression speed did not affect either the LMD position control or capacitive signal analysis under our experimental conditions.

We connected the electrodes with an LCR meter (4284A, Agilent) for capacitance determination, measured the switching device impedance while applying an AC voltage at 1.0 V and 1.0 MHz, and estimated the capacitance from the imaginary part of the impedance.

### Supplementary information

Video 1

Video 2

Video 3

Video 4

Supplementary information
