# Supplementary material for: Simultaneous detection of the shuttling motion of liquid metal droplets in channels under alternating pressure and capacitive sensor signals
Source: Microsyst Nanoeng. 2024 Mar 29;10:46. doi: 10.1038/s41378-024-00652-1 (PMC10978907; doi:10.1038/s41378-024-00652-1)
Supplement: Supplementary file 5 — Supplementary information [file 41378_2024_652_MOESM5_ESM.docx]

**Supplementary Information on the simultaneous detection of the shuttling motion of liquid metal droplets in channels under alternating pressure and capacitive sensor signals**

Shinji Bono^1,2,3*^, Ryotaro Nakai^4^, and Satoshi Konishi^1,2,3,4^

^1^ Research Organization of Science and Technology, Ritsumeikan University, Shiga, Japan

^2^ Ritsumeikan Advanced Research Academy, Kyoto, Japan

^3^ Ritsumeikan Global innovation Research Organization, Ritsumeikan University, Shiga, Japan

^4^ Graduate School of Science and Engineering, Ritsumeikan University, Shiga, Japan

* **Corresponding Author**

Email: [bono@fc.ritsumei.ac.jp](mailto:bono@fc.ritsumei.ac.jp)

**Video:**

Video 1–LMD in a channel with compression (MP4). The scale bar indicates 5 mm.

Video 2–LMD in a channel with decompression (MP4). The scale bar indicates 5 mm.

Video 3–LMD in the switching device under compression (MP4).

Video 4–LMD in the switching device under decompression (MP4).
